# Milk Beta-Hydroxybutyrate and Fat to Protein Ratio Patterns during the First Five Months of Lactation in Holstein Dairy Cows Presenting Treated Left Displaced Abomasum and Other Post-Partum Diseases

**DOI:** 10.3390/ani11030816

**Published:** 2021-03-14

**Authors:** Mariana Alves Caipira Lei, João Simões

**Affiliations:** 1 Department of Zootechnics, School of Agricultural and Veterinary Sciences, University of Trás-os-Montes and Alto Douro (UTAD), 5000-801 Vila Real, Portugal; marianalei@utad.pt; 2 Department of Veterinary Sciences, School of Agricultural and Veterinary Sciences, University of Trás-os-Montes and Alto Douro (UTAD), 5000-801 Vila Real, Portugal

**Keywords:** milk beta-hydroxybutyrate, fat to protein content ratio, left displaced abomasum, negative energy balance, postpartum diseases

## Abstract

**Simple Summary:**

This study aimed to evaluate the 5-month pattern (averaged days in milk; DIM1 to 5) of milk beta-hydroxybutyrate (BHB) concentration and fat to protein content (F:P) ratio patterns from Holstein cows presenting postpartum diseases which have been treated. Cows presenting left displaced abomasum (LDA) and concomitant diseases within the first three months had higher concentrations of BHB than the control group (cows without diseases) in the first, but not in the second month postpartum. The F:P ratio had a similar evolution pattern also for DIM2. Animals with LDA were four to six times more likely to have a F:P ratio ≥ 1.29 than the control group during DIM1 and DIM2, respectively. Moderate and high correlations were also observed between the F:P ratio and BHB in DIM1 and DIM2, respectively. We concluded that animals suffering from LDA within the first three months postpartum have a significantly higher concentration of BHB and F:P ratio in milk than cows without postpartum diseases during the first two months. The treated cows with LDA quickly recovered normal levels, up to DIM3. The F:P ratio is a viable and economic indicator, mainly between the first two months postpartum, to estimate BHB concentration and energy balance in cows presenting LDA and in recovery.

**Abstract:**

The main objective of the present study was to evaluate the beta-hydroxybutyrate (BHB) and fat to protein content (F:P) ratio patterns in the milk of Holstein cows with postpartum diseases throughout the first five months of lactation. This prospective study was performed at Vestjyske Dyrlaeger ApS (Nørre Nebel, Denmark). The milk fat, protein, and BHB were evaluated in the Danish Eurofins laboratory according to the monthly averaged days in milk (DIM1 to 5). According to clinical records, five groups were formed: A (control group; cows without diseases; *n* = 32), B (cows with left displaced abomasum -LDA- and concomitant diseases; *n* = 25); C (cows with other diseases up to DIM3; *n* = 13); D (cows with foot disorders up to DIM3; *n* = 26); and E (cows with disease manifestations in DIM4 and DIM5; *n* = 26). All the sick cows were treated after diagnosis, and laparoscopy was performed on cows with LDA. In group B, a higher concentration of BHB (0.18 ± 0.02 mmol/L; *p* < 0.001) was observed than in the control group (0.07 ± 0.02 mmol/L; *p* < 0.001) in DIM1, presenting an odds ratio (OR) = 8.9. In all groups, BHB decreased to 0.03–0.05 mmol/L (*p* < 0.05) since DIM3. The F:P ratio was higher in group B (1.77 ± 0.07) than in group A (1.32 ± 0.06; *p* < 0.05) in DIM1. A similar profile is observed in DIM2. It was observed that animals in group B were four to six times more likely to have a F:P ratio ≥1.29 during DIM1 (OR = 4.0; 95% CI:1.3–14.4; *p* = 0.01) and DIM2 (OR = 5.9; 95% CI %:1.9–21.9; *p* < 0.01), than cows in group A. There were also moderate and high correlations between the F:P ratio and the BHB for DIM1 (r = 0.57; r^2^ = 0.33; RSD = 0.09; *p* < 0.001) and DIM2 (r = 0.78; r^2^ = 0.60; RSD = 0.07; *p* < 0.001), respectively. We concluded that animals affected by LDA in the postpartum period have a higher concentration of BHB in milk in DIM1 and all treated animals quickly recover BHB levels up to DIM3. The F:P ratio is a viable and economic indicator, mainly in DIM1 and DIM2, to estimate BHB concentration and energy balance in cows with LDA and other postpartum diseases.

## 1. Introduction

The presence of high concentrations of ketone bodies in the blood—namely beta-hydroxybutyrate (BHB), acetoacetate, and acetone—is called hyperketonemia or ketosis and is one of the most harmful and damaging metabolic diseases in dairy cows, resulting from negative energy balance (NEB), especially during early lactation [1]. In Europe, a prevalence of subclinical ketosis has been reported (serum BHB ≥ 1.2 mmol/L) between 11.2 and 36.6% from 2nd to 15th days in milk (DIM) [2], similar to that observed in other continents, and where the prevalence of cows developing serum BHB ≥ 3.0 mmol/L, and related to clinical ketosis, was 3.4% on average [3].

Clinical ketosis, displacement of the abomasum (DA), metritis, and lameness are more likely to occur in dairy cows with hyperketonemia levels [2]. It is reasonable to expect that metabolic (oxidative) stress NEB at the beginning of lactation associated with hyperketonemia, will have a depressive effect on the immune system of the affected animals, increasing their susceptibility to the occurrence of pathologies such as metritis and laminitis, in this initial phase [2,4]. Moreover, Holstein cows, as other breeds such as Jerseys, Brown Swiss, Guernseys, Ayrshires, and Simmental-Red Holsteins commonly present DA [5]. Left side DA (LDA), with heritability estimated at 0.30 on Holstein cows [5], is more frequent than right side DA with implications for animal health and welfare, representing significant financial losses for dairy farmers.

LDA is common in high-producing dairy cows, mainly during the first month of lactation [6]. A surgical approach is usually required to treat this condition. A one-step laparoscopy-guided abomasopexy (Christiansen modified technique) treatment of LDA is a minimally invasive technique that allows the confirmation of the LDA and the evaluation of eventual adhesions between the abomasum and the left abdominal wall or rumen. More importantly, it can be performed completely in a standing cow, without the need to put it in recumbency, and is easier and faster than two-step laparoscopy [7,8,9]. According to Wapenaar and Roberts (2017), the survival rate ranges from 73 to 88% in six months [10].

The determination, by spectrophotometry, of the BHB concentration has been considered the gold standard method [1]. However, in recent years, due to the high correlation between the concentration of BHB in blood and milk (about 10 times less concentration), this latter fluid has served as a viable sample to determine this metabolite [11,12], being of common use in dairy farms. Another indirect method of assessing the energy balance is the fat to protein content (F:P) ratio [13]. An increase in the F:P ratio coincides with periods of NEB associated with increased mobilization of lipid reserves, which stimulates hyperketonemia and the fat content of milk [14]. According to Heuer et al. F:P ratio threshold between 1.35 and 1.50 can predict cows in energy deficit [13], but these values are not consensual [15]. Moreover, the F:P ratio of milk reflects the health status of the cow if the cow has already suffered or is suffering from the effects of a disease, so it can potentially serve as an indicator of energy deficit, including estimating hyperketonemia [16] and for monitoring energy balance during the first few months after delivery. Furthermore, van Knegsel et al. [17] and Denis-Robichaud et al. [18] suggested a F:P ratio threshold of ≥1.5 and ≥1.3 [18], respectively, to predict cows with hyperketonemia. The serum BHB concentration was used in these last studies.

Although numerous studies on the association between hyperketonemia and postpartum diseases, such as some of the above, have been carried out, we have not found relevant studies investigating the metabolic pattern of BHB measured by milk, or that of the F:P ratio preceding and following its treatment. The present study’s main objective is to determine the profiles of BHB in milk and the F:P ratio, during the first five months of lactation, in laparoscopic-treated LDA Holstein dairy cows. Other general postpartum diseases were also assessed.

## 2. Materials and Methods

### 2.1. Animals and Study Design

One hundred and twenty-two Holstein cows, at first (*n* = 43), second (*n* = 33), and third (*n* = 46) lactation, belonging to 20 dairy farms assisted by the veterinary clinic Vestjyske Dyrlaeger ApS (Denmark) were considered in the present prospective study. All animals calved between June 2019 and March 2020. All farms were managed for balanced diets.

Initially, we selected cows that presented LDA up to the first three months of lactation and that were treated by one-step laparoscopy-guided abomasopexy technique [7,8,9].

To create the control group, for each of the previously selected animals, between 1 and 4 animals from the same farm were selected to ensure equal environmental, nutritional, and management effects. In each holding, each pair or group pre-filled, cumulatively, the following requirements:

1. The same number of lactations;
2. Approximately the same date of delivery (±10 days) or the same DIM (±10 days). Cows were moved to the maternity unit 7 to 10 days before giving birth; so cows from each pair, who at some point during this period shared this park, were under the same effect of environmental and management factors;
3. Be under the same nutritional and management plan;
4. Present similar body condition.

In some cases of 2nd or 3rd lactation, the animals—for which at least one pair was not found with the same number of lactations—were paired with animals of 3rd or 2nd lactation, respectively, which met the remaining selection and matching criteria cumulatively.

In farms, a composite milk sample of each animal was collected from the respective milking machine or robot in a 30 mL container, without preservatives, which was previously prepared and identified by barcodes. The samples were immediately refrigerated and sent to the laboratory by CKR transporter (Aarhus, Denmark; see https://www.ryk-fonden.dk/). All lactating cows were sampled monthly with an interval as close to 30–31 days as possible, for the first five months throughout the study period, and according to the farms’ milk control management. The averaged DIM was calculated for each month according to the sequential milk samples collection and classified as DIM1 to DIM5. The milk BHB, protein, and fat were measured using Fourier-transform infrared spectroscopy (FTIR) methods at Eurofins Steins Laboratorium (Vejen, Denmark; see https://www.eurofins.dk/). Finally, data of milk production, percentages of fat and protein in milk, and BHB concentration in milk, as well somatic cells count, were assessed from test-day records using the DMS Dyreregistrering platform. All complete records (*n* = 610) were considered.

With the animals gathered for this study, and recording the clinical evolution during the five months postpartum, five different groups were formed:

- Group A: control group, i.e., cows without any apparent pathology during the 5 months of study (*n* = 32).
- Group B: cows with LDA (*n* = 25). The proportion of cows with LDA observed in DIM1 (40%) was similar to DIM2 (56%; *p* > 0.05) and greater than in DIM3 (4%; *p* < 0.001). In these animals, other pathologies (i.e., comorbidities) were observed, namely lameness (*n* = 22), clinical ketosis (*n* = 11; 6 and 5 in DIM1 and DIM2, respectively; *p* > 0.05), mastitis (*n* = 6), inflammation of unknown origin (*n* = 3), metritis (*n* = 6), digestive or respiratory disease (*n* = 3), hypocalcemia (*n* = 2), and uterine torsion (*n* = 1). It was observed that 33.3% (18/54), 38.9% (21/54), and 27.8% (15/54) of these diseases occurred in DIM1, DIM2, and DIM3, respectively (*p* > 0.05). Despite LDA, all cows of this group suffering lameness also presented one of the above-reported disease. All diseases were treated.
- Group C: cows with other diseases (that not LDA) up to third milk control (*n* = 13). The treated diseases were metritis (*n* = 6), lameness (*n* = 6), mastitis (*n* = 5), hypocalcemia (*n* = 2), and clinical ketosis (*n* = 1). It was observed that 60% (12/20; *p* < 0.001), 15% (3/20), and 25% (5/20) of the diseases occurred at DIM1, DIM2, and DIM3, respectively.
- Group D: cows with only foot disorders up to third milk control (*n* = 26). The distribution of foot problems was 15.4%, 42.3%, and 42.3% at DIM1, DIM2, and DIM3, respectively (*p* = 0.06)
- Group E: cows with disease manifestations only in fourth and fifth milk control (*n* = 26). The treated diseases were lameness (*n* = 20), mastitis (*n* = 4), pneumonia (*n* = 3), and inflammation of unknown origin (*n* = 1). Similar proportions (50%) occurred in DIM4 and DIM5.

### 2.2. Statistical Analysis

Differences in disease proportions between groups were evaluated using the chi-square test.

One-way ANOVA was used to evaluate averaged DIM differences between groups or between monthly milk sample collections.

ANOVA with repeated measures was used, based on the repetition of the milk samples through the software StatView^®^ 5.0 for Windows (SAS Institute, Cary, NC, USA), according to the following linear model:

y_ij_= μ + α_i_ + β_j_ + (αβ)_ij_ + ϵ_ijk_ (1)

where, y_ij_ is the value obtained from milk yield, milk BHB, milk protein, milk fat, or F/P ratio, μ is the overall mean, αi is the fixed effect of the ith level of group (i = A, B, C, D, E), β_j_ is the repeated effect jth level of DIM (j = 1, 2, 3, 4, 5), (αβ)_ij_ is the ij interaction effect and ϵ_ijk_ is the random error.

The Bonferroni/Dunn post hoc test was used to evaluate differences between pairs from one way ANOVA analysis and all models.

A receiver operating characteristic (ROC) curve was made to estimate F:P ratio threshold to detect cows with ≥0.14 mmol/L BHB, i.e., cows presenting ketosis according to Renaud et al. [12]. The odds ratio (OR) involving these thresholds were evaluated using the Wald test. Pearson’s correlations between BHB concentration and F:P ratio were also assessed. The software JMP^®^ 14 for Windows (SAS Institute, Cary, NC, USA) was used for these last purposes.

The results were presented as mean ± SD (standard deviation). The level of statistical significance for all results was *p* < 0.05.

## 3. Results

No differences of the averaged DIM were observed between groups (*p* > 0.05) in each sampling period when milk was successively sampled each month (DIM1: 20.1 ± 12.5; DIM2: 56.5 ± 19.7; DIM3: 89.1 ± 26.1; DIM4: 119.5 ± 25.9; DIM5: 155.2 ± 26.0 days; *p* < 0.001). The descriptive analysis of milk yield and milk contents is reported in Table 1.

### 3.1. Milk Yield

Contrarily to DIM (*p* < 0.05), no effect of groups was observed on the milk yield (*p* > 0.05). A higher milk yield was observed in DIM2 (37.9 ± 9.9 kg) than in DIM5 (35.9 ± 8.1 kg). Nevertheless, group × DIM interaction (*p* < 0.01) was also observed: group B had lower milk yield (33.7 ± 13.4 kg) than group E (41.5 ± 11.4 kg; *p* < 0.05), but only on DIM2.

### 3.2. BHB Concentration

Significant differences (*p* < 0.001) of BHBA were observed between groups, DIM and groups × DIM interaction. According to Table 2, a consistent decrease of BHBA during the first (group C) or second (group B) months were observed.

No significant difference in milk BHB concentration was observed between DIM1 (0.20 ± 0.06 mmol/L) and DIM2 (0.22 ± 0.06 mmol/L; *n* = 14; *p* = 0.60) in cows presenting LDA in DIM2. Nevertheless, half of the cows (*n* = 5) suffering LDA in DIM1 also presented high milk BHB concentration in DIM2 (0.11 ± 0.03 mmol/L; *n* = 5).

### 3.3. Fat Content in Milk

Significant differences were found between groups (*p* < 0.01) and DIM (*p* < 0.001), and group × DIM interaction (*p* < 0.001) on milk fat as reported in Table 3.

### 3.4. Protein Content in Milk

There were no significant differences (*p* = 0.92) in the protein between groups. However, there was an effect of the contrast days (*p* < 0.001). The interaction between the two variables was not significant (*p* = 0.30; Table 4).

### 3.5. Fat to Protein Content Ratio

An effect of groups (*p* < 0.01), DIM and groups (*p* < 0.001) × DIM interaction (*p* < 0.001) on the F:P ratio was also observed (Table 5).

### 3.6. Relationships between Beta-Hydroxybutyrate and Fat to Protein Content Ratio or Milk Yield/Contents

**Figure 1:**
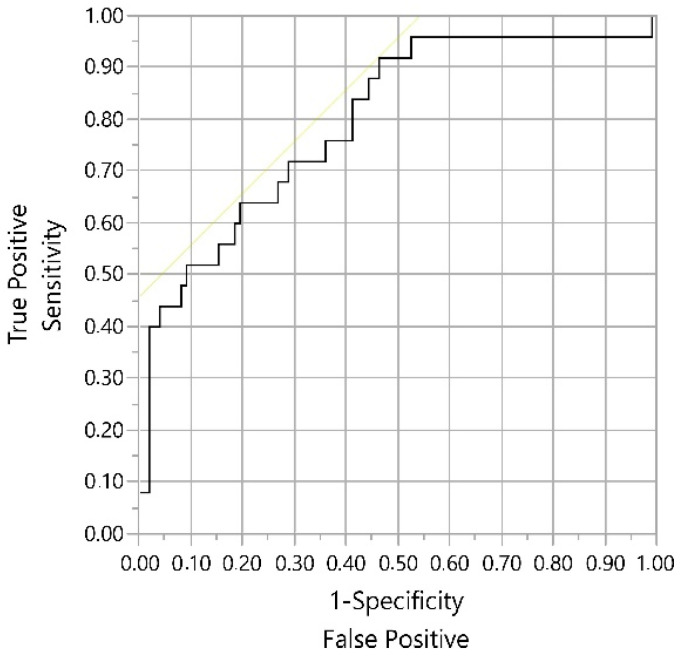
Receiver operating characteristic (ROC) curve to calculate the threshold ratio F:P to predict hyperketonemia (subclinical ketosis). Positive level: beta-hydroxybutyrate ≥ 0.14 mmol/L; *p* < 0.001; AUC (area under the curve) = 0.80.

It was observed a F:P ratio threshold ≥1.29 to estimate cows with ≥0.14 mmol/L BHB, according to the ROC curve (Figure 1) in DIM1. The sensitivity and specificity were 92.0% and 54%, respectively. Similar values were obtained when overall DIM (1 to 5) were considered.

Group B animals were four to six times more likely to have a F:P ratio ≥1.29 than group A cows, in DIM1 and DIM2, respectively (Table 6). Moreover, this group showed about nine times greater chance than group A (control) of presenting hyperketonemia (BHB ≥ 0.14 mmol/L) in DIM1, but not in the remaining periods. Group C animals did not show significant differences in any of the periods.

Moderate and high correlations were observed between the F:P ratio or milk fat and BHB for DIM1 and DIM2 (Table 7).

## 4. Discussion

Cows with LDA (group B) were found to have higher BHB concentrations in milk (0.18 ± 0.02 mmol/L) than the control group (0.07 ± 0.02 mmol/L; *p* < 0.001) in DIM1, decreasing rapidly in the following month, even though the proportion of LDA and clinical ketosis was similar in DIM1 and DIM2. The results also show that the animals recover quickly after treatment, with no significant differences in BHB between groups from DIM3.

The control group kept BHB levels (0.7 mmol/ L) in DIM1 and DIM2 stable, which halved in DIM3, when the peak dry matter intake was reached, indicating that the energy management of the farms in question was adequate to the different groups. The higher concentrations of BHB at DIM1 and DIM2 of group A (control) follow the same trend found by Belay et al. [19]; these authors observed that milk BHB concentrations (obtained by FTIR technique) were higher between 11–61 DIM [19]. Previously, Koeck et al. had already verified the same trend [20]. This is probably due to the increase in dry matter intake and the gradual return to the positive energy balance as the days in milk progress [21]. It is known that the manifestation of clinical ketosis is not necessarily associated with higher serum concentrations of BHB [2] and that the lower capacity of dry matter intake, and as a consequence, lower supply of energy, as well as the process of adaptation of the papillae and rumen microbiome, may have influenced the greater degree of energy deficit in this phase (DIM1).

Although group C did not show any significant difference with the control group in DIM1, a similar profile of decrease in BHB was observed, with significant differences in DIM3. Unlike group B, whose distribution of LDA as well as their concomitant pathologies, were homogeneous (*p* > 0.05) in DIM1 and DIM2, in group C, 60% of the pathologies occurred in DIM1 (*p* < 0.001), with no significant differences to group A or group B. This suggests that although there may be an additive effect of disease association, this effect is largely dependent on the time that has elapsed since the beginning of lactation. The relation of pathologies associated with the beginning of lactation, and the position they occupy in a cause-effect relationship, are complex issues and not yet fully clarified [1]. Most authors believe that cows with ketosis have an increased risk of developing other pathologies at the beginning of lactation [1]. Effectively, Raboisson et al. summarized from multiple studies that the OR of hyperketonemic animals develop different pathologies: 5.4 (3.3–8.8) for clinical ketosis; 3.3 (2.6–4.3) for abomasum displacement; 1.8 (1.5–2.0) for metritis; 1.5 (1.2–1.9) for placental retention; 1.6 (1.2–2.1) for mastitis; 1.4 (1.3–1.6) for somatic cells count duplication; 2.0 (1.6–2.4) for laminitis, and 1.9 (1.6–2.3) for early culling [22]. This same risk, according to McArt et al. [23] and Suthar et al. [2], increases with increasing blood BHB concentration. McArt et al. [23] observed that cows diagnosed with ketosis from the third to the fifth day of lactation were 6.1 (95% CI = 2.3 to 16.0) times more likely to develop displacement of the abomasum than cows diagnosed after the first week. Ketosis probably induces hypoglycemia in multiparous cows [24] and decreased time and rumination activity [25]. Duffield et al. [26] observed that some cows developed pathologies, such as DA, before being diagnosed with ketosis, reinforcing the hypothesis that the pathologies associated with ketosis may be more than the effects of this, the cause. The interaction between ketosis and the DA has been stated as bidirectional, proposing that they can both be a risk factor or a consequence of each other. Inclusively they are both multifactorial diseases and there are already described some risk factors that are common to both pathologies, mostly related to housing, management, and feeding adopted systems [27]. Suthar et al. [2] recorded both diseases at the same time and verified that they were both related to high levels of BHB, proving that they are both directly correlated with NEB. Duffield et al. [26] hypothesized the explanation: the presence of common factors of hyperketonemia and DA with a similar causal etiologic is also related to a poor adaptive response at the onset of lactation conducting to NEB. This same theory has already been addressed regarding other postpartum diseases like mastitis and metritis [28].

In our study, only 11 of the 25 cows with LDA had clinical ketosis in the same month that the pathology was diagnosed, which shows the complexity of demonstrating cause and effect. An interesting finding is the observed decrease of the BHB concentration from DIM1 to DIM2, DIM2 as the period with the higher number of LDA (14 LDA). Duffield et al. [26], McArt et al. [23] and Suthar et al. [2] suggested that ketosis is a risk factor for LDA; It would be expected that the month with the higher number of diagnosed LDA (DIM2 in our study) presents high concentration of BHB, and the OR increases with increasing BHB concentration. In the present study, similar BHB concentrations of the animals that suffered from LDA on DIM2 were observed between DIM1 and DIM2 suggesting that, in these cases, ketosis contributed to LDA etio-pathophysiology. Nevertheless, BHB concentrations remained high in DIM2 for cows suffering LDA in DIM1 suggesting that hyperketonemia could be a consequence of LDA. These findings are in agreement with the bidirectional association between ketosis and LDA referred to in the Stengärde et al. study [27].

We observed that the fat content in milk in the different groups was maximum at the first milk control, a control that mirrors the repercussions of the NEB that can be installed up to three weeks postpartum. Furthermore, and in agreement with Zhang et al. [14], in our study, the maximum percentages of fat content in milk also correspond to the maximum concentrations of BHB in milk for most groups, as we can see through the higher fat content in group B than in group A. Additionally, moderate and higher correlations between fat content in milk and BHB in group B were observed in DIM1 and DM2, respectively. From the second or third milk control, after the NEB, we saw a less abrupt decrease in the fat content in milk in all groups.

During phases of energy deficit, such as the beginning of lactation, there are increased levels of fat content in milk [15] and decreased levels of protein [29]. The high concentrations of fat content in milk in periods of NEB correspond in more than 95% to triglycerides (TG) [14] and are mainly due to the increased mobilization of lipid reserves [15] that lead to the production of a large amount of BHB [30] that induces increased synthesis of TG by mammary epithelial cells [14].

The essential fatty acids to the synthesis of these TG come from the diet or the activity of the ruminal microbiota [31]. In the mammary gland, there is also the “de novo” synthesis in the mammary epithelial cells. It is in the “de novo” synthesis that BHB, mainly from the butyric acid metabolization, gains special importance; in this way, in ruminants, BHB is one of the main precursors (originating about 50%) of the “de novo” synthesized fat in mammary epithelial cells [14].

Regarding the protein content in milk, we observed that there was a decrease between DIM1 and DIM2 for groups A and E, contrary to what was observed in groups B, C, and D in which diseases occur in the first three months of lactation. The decrease in protein content in milk is a normal situation, since NEB worsens by that time, reducing milk protein synthesis. These data are in agreement with the conclusions of Gross et al. [15] that protein evolves inversely to milk productivity and hence it is expected that, at the beginning of lactation when productivity is low, the maximum levels of protein in milk can be verified. In fact, a healthy animal reflects the expected start of lactation and therefore relatively low productivity that increases until the peak of lactation.

The non-decreasing in milk protein in groups B, C, and D, between DIM1 and DIM2, may be related to the lower protein supply in DIM1 due to the potential lower DMI that normally occurs during the manifestation of diseases (anorexia). A significant part of these groups developed some form of the disease during the first month. In some of these cases, there may still be less protein production in milk due to the worsening of NEB, since the increase in circulating non-esterified fatty acids may inhibit the production of somatotropin (growth hormone; Akers) [32]. It should be noted that the amount of milk was not significantly reduced in these groups (except between groups B and E at DIM2) at the time of sample collection, which due to the normal lag between the occurrence of the disease and that the moment of milk sample collection indicates that this production is quickly resumed after treatment.

The amount of fat and protein reflects a cow’s energy status/condition [15]. Toni et al. [33] observed that cows with high F:P ratios at the beginning of lactation showed a higher incidence of various pathologies and were, as a result, more often early culled. Similar to the results found by Toni et al. [33], we observed that animals affected by LDA (group B) and various pathologies other than LDA (group C), presented higher F:P ratios in DIM1 than cows in the control group. Furthermore, we found that animals in group B with a F:P ratio ≥1.29 are about four to six times more likely to have BHB concentrations ≥0.14 mmol/L, that is, of having subclinical ketosis. These observations are accompanied by moderate and high correlations between the F:P ratio and the BHB for DIM1, in this same group. Zhang et al. [14] observed, in vitro, that animals with high circulating concentrations of BHB had higher TG content, indicating that high concentrations of BHB increase TG synthesis by mammary epithelial cells, justifying why animals with ketosis generally have milk with higher fat content. The increase in the F:P ratio, therefore, coincides evidently with periods of NEB associated with increased mobilization of lipid reserves and consequently with the increase in circulating levels of BHB.

Our results indicate that the determination of the F:P ratio, starting at 1.29, is a useful tool in the management of dairy cattle health, allowing the estimation of subclinical ketosis associated with LDA and other postpartum pathologies with high sensitivity and reasonable specificity. This F:P ratio threshold agrees with the ≥1.3 threshold obtained by Denis-Robichaud et al. [18] to predict hyperketonemia (serum BHB ≥ 1.4 mmol/L). Equally important, the present study emphasizes that in farms that offer, to their animals, diets with correctly balanced energy, cows with health problems (mainly LDA) are the ones most associated with the development of ketosis and most exposed to greater negative energy balance during the first two months of lactation, especially during the first month. Therefore, cows with high BHB and F:P ratio in milk in DIM1 should be preventively monitored for the diagnosis of the pathologies in question (groups B and C) in the first two months. This strategy allows a timely (earlier) diagnosis and respective treatment. Cows with diseases on the third, fourth, and fifth months of lactation do not have such a significant impact on the evaluated milk parameters (subclinical ketosis and NEB).

## 5. Conclusions

In conclusion, animals with LDA and/or other pathologies of the beginning of lactation are the ones that should be monitored and require more attention, concerning the BHB concentration, mainly in the first two milk controls. These animals, after treatment, recovered from LDA episodes and showed levels of BHB and other metabolites identical to healthy animals. Our work also suggests that lameness does not significantly influence concentrations of BHB or F:P ratio.

The determination of the F:P ratio profile during the postpartum period seems to be an important and economic indicator that can complement the evaluation of animals affected with LDA and even other postpartum pathologies. Special attention should be given to high-yielding cows presenting a F:P ratio ≥ 1.29 which can indicate the presence of a more intense NEB (≥0.14 mmol/L BHB) related to LDA.

## Figures and Tables

**Table 1:** Descriptive analysis of milk yield and milk contents from all 610 milk samples.

Milk Parameter	Mean ± SD	95% Confidence Interval
Milk yield (kg)	37.0 ± 9.5	36.2–37.7
Fat (%)	4.09 ± 0.93	4.01–4.16
Protein (%)	3.41 ± 0.29	3.39–3.43
BHB (mmol/L)	0.06 ± 0.04	0.05–0.06
SCC (10^3^/mL) ^1^	236.5 ± 552.6	192.6–280.5

^1^ Arithmetic mean. SD: standard deviation; BHB: beta-hydroxybutyrate; SCC: somatic cells count.

**Table 2:** BHB concentration (mmol/L) pattern by group over the first five months of lactation.

Group	DIM1	DIM2	DIM3	DIM4	DIM5
A	0.07 ± 0.02 ^A,a^	0.06 ± 0.02 ^a^	0.03 ± 0.01 ^b^	0.03 ± 0.01 ^b^	0.04 ± 0.01 ^a,b^
B	0.18 ± 0.02 ^B,a^	0.12 ± 0.02 ^a,b^	0.05 ± 0.01 ^b^	0.04 ± 0.01 ^b^	0.04 ± 0.01 ^b^
C	0.14 ± 0.03 ^A,B,a^	0.07 ± 0.03 ^b^	0.04 ± 0.01 ^b^	0.04 ± 0.01 ^b^	0.04 ± 0.01 ^b^
D	0.06 ± 0.02 ^A^	0.06 ± 0.02	0.05 ± 0.01	0.04 ± 0.01	0.03 ± 0.01
E	0.06 ± 0.02 ^A^	0.04 ± 0.02	0.04 ± 0.01	0.04 ± 0.01	0.05 ± 0.01

DIM: Days in milk. ^A,B^ different letters within the same column: *p* < 0.001. ^a,b^ different letters within the same line: *p* < 0.05.

**Table 3:** Averaged milk fat (%) pattern by group over the first five months of lactation.

Group	DIM1	DIM2	DIM3	DIM4	DIM5
A	4.59 ± 0.22 ^A,a^	3.89 ± 0.14 ^A,b^	3.76 ± 0.10 ^b^	1.2 ± 0.11 ^b^	3.76 ± 0.09 ^b^
B	5.98 ± 0.25 ^B,a^	4.54 ± 0.16 ^B,b^	3.86 ± 0.12 ^b^	1.2 ± 0.12 ^b^	3.92 ± 0.10 ^b^
C	5.20 ± 0.34 ^A,B,a^	4.06 ± 0.22 ^A,B,b^	3.80 ± 0.16 ^b^	1.2 ± 0.17 ^b^	3.59 ± 0.15 ^b^
D	4.51 ± 0.24 ^A,a^	3.99 ± 0.16 ^A,a,b^	3.81 ± 0.12 ^b^	1.13 ± 0.12 ^b^	3.87 ± 0.10 ^a,b^
E	4.66 ± 0.24 ^A,a^	3.83 ± 0.16 ^A,b^	3.80 ± 0.12 ^b^	1.07 ± 0.12 ^b^	3.66 ± 0.10 ^b^

DIM: days in milk. ^A,B^ different letters within the same column: *p* < 0.05. ^a,b^ different letters within the same line: *p* < 0.05.

**Table 4:** Milk protein content in milk (%) pattern by group over the first five months of lactation.

Group	DIM1	DIM2	DIM3	DIM4	DIM5
A	3.49 ± 0.40 ^a^	3.27 ± 0.20 ^b^	3.39 ± 0.21 ^a,b^	3.44 ± 0.21 ^a,b^	3.46 ± 0.21 ^a^
B	3.39 ± 0.43 ^a,b^	3.26 ± 0.29 ^a^	3.40 ± 0.26 ^a,b^	3.471 ± 0.23 ^a,b^	3.50 ± 0.22 ^b^
C	3.50 ± 0.42	3.23 ± 0.28	3.30 ± 0.23	3.40 ± 0.23	3.48 ± 0.24
D	3.41 ± 0.32 ^a,b^	3.26 ± 0.27 ^a^	3.33 ± 0.25 ^a,b^	3.43 ± 0.25 ^a,b^	3.47 ± 0.24 ^b^
E	3.60 ± 0.40 ^a^	3.26 ± 0.20 ^b^	3.31 ± 0.26 ^b^	3.45 ± 0.25 ^a,b,c^	3.52 ± 0.26 ^a,c^

DIM: days in milk. ^a,b,^^c^ different letters within the same line: *p* < 0.05.

**Table 5:** F:P ratio pattern by group over the first five months of lactation.

Group	DIM1	DIM2	DIM3	DIM4	DIM5
A	1.32 ± 0.06 ^A,a^	1.19 ± 0.05 ^A,b^	1.19 ± 0.03 ^b^	1.09 ± 0.03 ^b^	1.09 ± 0.02 ^b^
B	1.77 ± 0.07 ^B,a^	1.41 ± 0.06 ^B,b^	1.14 ± 0.04 ^b,c^	1.13 ± 0.04 ^c^	1.12 ± 0.03 ^c^
C	1.47 ± 0.09 ^A,B,a^	1.26 ± 0.08 ^A,B,a,b^	1.15 ± 0.05 ^b^	1.06 ± 0.05 ^b^	1.05 ± 0.04 ^b^
D	1.33 ± 0.06 ^A,a^	1.23 ± 0.06 ^A,B,a,b^	1.15 ± 0.03 ^b^	1.13 ± 0.03 ^b^	1.14 ± 0.03 ^b^
E	1.29 ± 0.06 ^A,a^	1.18 ± 0.05 ^A,a,b^	1.15 ± 0.03 ^b^	1.07 ± 0.03 ^b^	1.11 ± 0.03 ^b^

DIM: days in milk. ^A,B^ different letters within the same column: *p* < 0.05. ^a,b,c^ different letters within the same line: *p* < 0.05.

**Table 6:** The odds ratio for group B to detect cows with fat to protein content ratio (F:P ratio) ≥ 1.29 or beta-hydroxybutyrate levels ([BHB]) ≥ 0.14 mmol/L (group A as reference).

Parameter	DIM	Odds Ratio	95% CI
F:P ratio ≥ 1.29	1	4.0 **	1.3–14.4
	2	5.9 **	1.9–21.9
BHB ≥ 0.14 mmol/L	1	8.9 ***	2.6–37.2

DIM: days in milk; 95% CI: 95% confidence interval; **: *p* < 0.01; ***: *p* < 0.001.

**Table 7:** Correlations and regression equations between milk yield, milk contents, or fat to protein content ratio (F:P ratio) and beta-hydroxybutyrate (BHB).

Correlation ^1^ for (X):	DIM	r	r^2^	Regression Equation (Y = BHB/mmol)	RSD
Milk yield (kg)	2	0.24 **	0.06	Y = −0.038 + 0.003 × X (kg)	0.11
Milk fat (%)	1	0.46 ***	0.21	Y = −0.085 + 0.037 × X (%)	0.09
	2	0.71 ***	0.50	Y = −0.331 + 0.099 × X (%)	0.08
Milk protein (%)	2	−0.25 **	0.06	Y = 0.469 – 0.122 × X (%)	0.11
	3	−0.35 ***	0.13	Y = 0.286 – 0.072 × X (%)	0.05
	4	−0.38 ***	0.14	Y = 0.219 – 0.053 × X (%)	0.03
	5	−0.28 **	0.08	Y = 0.173 – 0.038 × X (%)	0.07
F:P ratio	1	0.57 ***	0.33	Y = −0.136 + 0.163 × X	0.09
	2	0.78 ***	0.60	Y = −0.322 + 0.314 × X	0.07

^1^ The correlations of the omitted DIM for each parameter were not significant (*p* > 0.05). DIM: days in milk; r: correlation coefficient; regression coefficient; RSD: residual standard deviation; **: *p* < 0.01; ***: *p* < 0.001.

## Data Availability

Data used in the present study can be requested to Vestjyske Dyrlaeger ApS (Denmark).
